# Supplementary material for: Prediction of 7‐year's conversion from subjective cognitive decline to mild cognitive impairment
Source: Hum Brain Mapp. 2020 Oct 8;42(1):192–203. doi: 10.1002/hbm.25216 (PMC7721238; doi:10.1002/hbm.25216)
Supplement: Supplementary file 1 — Appendix S1: Supporting Information [file HBM-42-192-s001.docx]

**Supplementary materials**

**Supplementary Table 1**

**The 223 features used in the machine learning-based classification and their selection frequency**

| **Features** | **Selection frequency** |
| --- | --- |
| **Demographic informatio****n (features #1-6)** | |
| Gender  Age  Education (year)  Smoke (years)  Drink (years)  BMI (kg/m^2^) | 0 (0%)  2 (2.6%)  75 (98.7%)  0 (0%)  54 (71.1%)  47 (61.8%) |
| **Sociodemographic and health measures (features #7-21)** | |
| Hypertension at baseline (year)  Diabetes mellitus at baseline (year)  Hyperlipidemia at baseline (year) ^†^  Stroke history at baseline  Heart disease at baseline  Surgical history at baseline  Sleep disorder at baseline  Hypertension at follow-up (year)  Diabetes mellitus at follow-up (year)  Hyperlipidemia at follow-up (year)  Stroke history at follow-up  Heart disease at follow-up  Surgical history at follow-up  Sleep disorder at follow-up  Hearing loss at follow-up | 0 (0%)  4 (5.3%)  0 (0%)  76 (100%)  0 (0%)  0 (0%)  0 (0%)  1 (1.3%)  0 (0%)  0 (0%)  0 (0%)  0 (0%)  2 (2.6%)  3 (3.9%)  23 (30.3%) |
| **Neuropsychological tests (features #22-25)** | |
| Geriatric Depression Scale (GDS) at baseline  Social support score at baseline  Montreal Cognitive Assessment (MoCA) at baseline  GDS at follow-up | 0 (0%)  0 (0%)  76 (100%)  22 (28.9%) |
| **^a^Brain imaging** **(features #26-223)** | |
| **A. Global biomarkers (features #26-71)** | |
| EstimatedTotalIntraCranialVol  CSF  TotalGrayVol  rhCerebralWhiteMatterVol  lhCerebralWhiteMatterVol  CerebralWhiteMatterVol  RightCerebellumWhiteMatter  LeftCerebellumWhiteMatter  WMhypointensities  BrainStem  SubCortGrayVol  CortexVol  BrainSegVol  BrainSegVolNotVent  BrainSegVolNotVentSurf  SupraTentorialVol  SupraTentorialVolNotVent  SupraTentorialVolNotVentVox  SurfaceHoles  OpticChiasm  MaskVol  LeftUnsegmentedWhiteMatter  RightUnsegmentedWhiteMatter  BrainSegVoltoeTIV  MaskVoltoeTIV  3rdVentricle  4thVentricle  RightCerebellumCortex  LeftCerebellumCortex  RightInfLatVent  LeftInfLatVent  Rightchoroidplexus  Leftchoroidplexus  Rightvessel  Leftvessel  rhCortexVol  lhCortexVol  rhSurfaceHoles  lhSurfaceHoles  RightLateralVentricle  LeftLateralVentricle  CC_Anterior  CC_Central  CC_Mid_Anterior  CC_Mid_Posterior  CC_Posterior | 0 (0%)  0 (0%)  0 (0%)  0 (0%)  0 (0%)  0 (0%)  0 (0%)  0 (0%)  47 (61.8%)  0 (0%)  0 (0%)  14 (18.4%)  0 (0%)  0 (0%)  0 (0%)  0 (0%)  0 (0%)  0 (0%)  0 (0%)  0 (0%)  0 (0%)  7 (9.2%)  21 (27.6%)  0 (0%)  0 (0%)  0 (0%)  0 (0%)  0 (0%)  0 (0%)  0 (0%)  1 (1.3%)  0 (0%)  0 (0%)  0 (0%)  0 (0%)  3 (3.9%)  2 (2.6%)  0 (0%)  0 (0%)  0 (0%)  0 (0%)  3 (3.9%)  8 (10.5%)  0 (0%)  0 (0%)  0 (0%) |
| **B. Subcortical gray matter volume** **(features #72-87)** | |
| RightHippocampus  LeftHippocampus  RightAmygdala  LeftAmygdala  RightPutamen  LeftPutamen  RightCaudate  LeftCaudate  RightPallidum  LeftPallidum  RightAccumbensarea  LeftAccumbensarea  RightThalamusProper  LeftThalamusProper  RightVentralDC  LeftVentralDC | 0 (0%)  4 (5.3%)  0 (0%)  76 (100%)  1 (1.3%)  0 (0%)  14 (18.4%)  0 (0%)  1 (1.3%)  0 (0%)  31 (40.8%)  0 (0%)  0 (0%)  0 (0%)  0 (0%)  13 (17.1%) |
| **C. 68 Cortical thickness (features #88-155)** | |
| Right bankssts  Right caudal anterior cingulate  Right caudal middle frontal  Right cuneus  Right entorhinal  Right frontal pole  Right fusiform  Right inferior parietal  Right inferior temporal  Right insula  Right isthmus cingulate  Right lateral occipital  Right lateral orbitofrontal  Right lingual  Right medial orbitofrontal  Right middle temporal  Right paracentral  Right parahippocampal  Right pars opercularis  Right pars orbitalis  Right pars triangularis  Right pericalcarine  Right postcentral  Right posterior cingulate  Right precentral  Right precuneus  Right rostral anterior cingulate  Right rostral middle frontal  Right superior frontal  Right superior parietal  Right superior temporal  Right supramarginal  Right temporal pole  Right transverse temporal  Left bankssts  Left caudal anterior cingulate  Left caudal middle frontal  Left cuneus  Left entorhinal  Left frontal pole  Left fusiform  Left inferior parietal  Left inferior temporal  Left insula  Left isthmus cingulate  Left lateral occipital  Left lateral orbitofrontal  Left lingual  Left medial orbitofrontal  Left middle temporal  Left paracentral  Left parahippocampal  Left pars opercularis  Left pars orbitalis  Left pars triangularis  Left pericalcarine  Left postcentral  Left posterior cingulate  Left precentral  Left precuneus  Left rostral anterior cingulate  Left rostral middle frontal  Left superior frontal  Left superior parietal  Left superior temporal  Left supramarginal  Left temporal pole  Left transverse temporal | 0 (0%)  0 (0%)  0 (0%)  0 (0%)  0 (0%)  0 (0%)  0 (0%)  0 (0%)  0 (0%)  0 (0%)  0 (0%)  0 (0%)  0 (0%)  0 (0%)  0 (0%)  0 (0%)  0 (0%)  0 (0%)  0 (0%)  0 (0%)  0 (0%)  0 (0%)  0 (0%)  0 (0%)  0 (0%)  0 (0%)  2 (2.6%)  0 (0%)  0 (0%)  0 (0%)  1 (1.3%)  0 (0%)  0 (0%)  0 (0%)  27 (35.5%)  0 (0%)  0 (0%)  0 (0%)  0 (0%)  0 (0%)  0 (0%)  0 (0%)  0 (0%)  2 (2.6%)  0 (0%)  0 (0%)  0 (0%)  0 (0%)  0 (0%)  0 (0%)  0 (0%)  0 (0%)  0 (0%)  0 (0%)  0 (0%)  0 (0%)  3 (3.9%)  0 (0%)  0 (0%)  0 (0%)  61 (80.3%)  0 (0%)  0 (0%)  0 (0%)  0 (0%)  0 (0%)  68 (89.5%)  0 (0%) |
| **D. 68 white matter region (features #156-223)** | |
| Right bankssts  Right caudal anterior cingulate  Right caudal middle frontal  Right cuneus  Right entorhinal  Right frontal pole  Right fusiform  Right inferior parietal  Right inferior temporal  Right insula  Right isthmus cingulate  Right lateral occipital  Right lateral orbitofrontal  Right lingual  Right medial orbitofrontal  Right middle temporal  Right paracentral  Right parahippocampal  Right pars opercularis  Right pars orbitalis  Right pars triangularis  Right pericalcarine  Right postcentral  Right posterior cingulate  Right precentral  Right precuneus  Right rostral anterior cingulate  Right rostral middle frontal  Right superior frontal  Right superior parietal  Right superior temporal  Right supramarginal  Right temporal pole  Right transverse temporal  Left bankssts  Left caudal anterior cingulate  Left caudal middle frontal  Left cuneus  Left entorhinal  Left frontal pole  Left fusiform  Left inferior parietal  Left inferior temporal  Left insula  Left isthmus cingulate  Left lateral occipital  Left lateral orbitofrontal  Left lingual  Left medial orbitofrontal  Left middle temporal  Left paracentral  Left parahippocampal  Left pars opercularis  Left pars orbitalis  Left pars triangularis  Left pericalcarine  Left postcentral  Left posterior cingulate  Left precentral  Left precuneus  Left rostral anterior cingulate  Left rostral middle frontal  Left superior frontal  Left superior parietal  Left superior temporal  Left supramarginal  Left temporal pole  Left transverse temporal | 76 (100%)  1 (1.3%)  0 (0%)  0 (0%)  1 (1.3%)  26 (34.2%)  0 (0%)  0 (0%)  24 (31.6%)  0 (0%)  0 (0%)  0 (0%)  0 (0%)  0 (0%)  0 (0%)  0 (0%)  0 (0%)  0 (0%)  0 (0%)  0 (0%)  0 (0%)  0 (0%)  0 (0%)  12 (15.8%)  0 (0%)  13 (17.1%)  0 (0%)  0 (0%)  0 (0%)  0 (0%)  0 (0%)  0 (0%)  0 (0%)  0 (0%)  0 (0%)  0 (0%)  0 (0%)  0 (0%)  0 (0%)  0 (0%)  0 (0%)  2 (2.6%)  22 (28.9%)  0 (0%)  0 (0%)  0 (0%)  0 (0%)  0 (0%)  0 (0%)  10 (13.2%)  0 (0%)  0 (0%)  0 (0%)  0 (0%)  0 (0%)  0 (0%)  0 (0%)  65 (85.5%)  0 (0%)  23 (30.3%)  0 (0%)  0 (0%)  0 (0%)  0 (0%)  0 (0%)  0 (0%)  3 (3.9%)  0 (0%) |

**^a^** For the name of the labeled brain regions, please refer to http://surfer.nmr.mgh.harvard.edu/fswiki/FsTutorial/anatomicalROI/FreesuferColorLU.

**Supplementary Table 2**

Confusion matrix for pSCD vs. sSCD classification (N = 76)

|  | **Predicted pSCD** | **Predicted sSCD** |
| --- | --- | --- |
| **Actual pSCD** | 15 | 9 |
| **Actual sSCD** | 14 | 38 |

**Supplementary Table 3**

Comparison of Different Feature Combination

| Features | accuracy | | sensitivity | specificity | F1 score |
| --- | --- | --- | --- | --- | --- |
| All | | 69.74% | 62.50% | 73.08% | 0.5660 |
| Psychological and clinical | | 56.58% | 41.67% | 63.46% | 0.3774 |
| MRI only | | 63.16% | 41.67% | 73.08% | 0.4167 |
| Psychological only | | 48.68% | 8.33% | 67.31% | 0.0930 |
| MRI and clinical | | 72.37% | 54.17% | 80.77% | 0.5532 |

Note: the “psychological” information refers to the neuropsychological test (features #22-25 in Supplementary Table 1); the “clinical” data refers to both demographic information, ociodemographic and health measures (features #1-21), and the “MRI” were from the Brain imaging (feature #26-223).

**Supplementary Table 4**

Comparison of the five selected contributing features between pSCD and sSCD

|  | **pSCD (N = 24)** | **sSCD (N = 52)** | ***p*** |
| --- | --- | --- | --- |
| Years of education | 7.17 (4.15) | 9.50 (3.06) | 0.019 |
| MoCA at baseline | 22.96 (4.49) | 24.04 (3.90) | 0.29 |
| Stroke history at baseline | 4 (16.7%) | 3 (5.8%) | 0.27 |
| Left amygdala’s volume | 2.73 (0.39) | 2.96 (0.33) | 0.011 |
| Right wmSTSbanks’ volume | 0.64 (0.11) | 0.55 (0.10) | 0.0005 |

Mean values with stand deviation or number with % are reported. NOTE: Independent-Samples *t* test or *Chi-*square test was used without correction. The region volume was standardized (no unit).

**Supplementary Table 5**

Comparison of the sMRI features between pSCD and sSCD

|  | **pSCD (N = 24)** | **sSCD (N = 52)** | ***p*** |
| --- | --- | --- | --- |
| EstimatedTotalIntraCranialVol (×10^3^mm^3^) | 1418.84 (147.58) | 1473.88 (154.90) | 0.15 |
| TotalGrayVol (×10^3^mm^3^) | 541.15 (44.63) | 568.81 (43.03) | 0.012 |
| SubCortGrayVol (×10^3^mm^3^) | 50.12 (4.55) | 52.61 (4.74) | 0.034 |
| CortexVol (×10^3^mm^3^) | 399.46 (34.84) | 422.96 (32.58) | 0.006 |
| Left bankssts thickness(×mm^3^) | 2.36 (0.14) | 2.40 (0.13) | 0.23 |
| Right bankssts thickness(×mm^3^) | 2.44 (0.13) | 2.45 (0.16) | 0.69 |
| CerebralWhiteMatterVol (×10^3^mm^3^) | 405.28 (55.39) | 428.03 (49.59) | 0.077 |
| Left wmSTSbanks (×10^3^mm^3^) | 2.5 (0.4) | 2.58 (0.53) | 0.52 |
| Right wmSTSbanks (×10^3^mm^3^) | 2.58 (0.5) | 2.35 (0.5) | 0.068 |
| Left superior temporal thickness(×mm^3^) | 2.50(0.12) | 2.59(0.14) | 0.007 |
| Right superior temporal thickness(×mm^3^) | 2.54(0.13) | 2.63(0.14) | 0.011 |

Mean values with stand deviation are reported. Note: Independent-sample *t* test was used without correction.

**Supplementary Figure** **1 |** The human right white matter of wmSTSbanks (purple) and left amygdala (green)


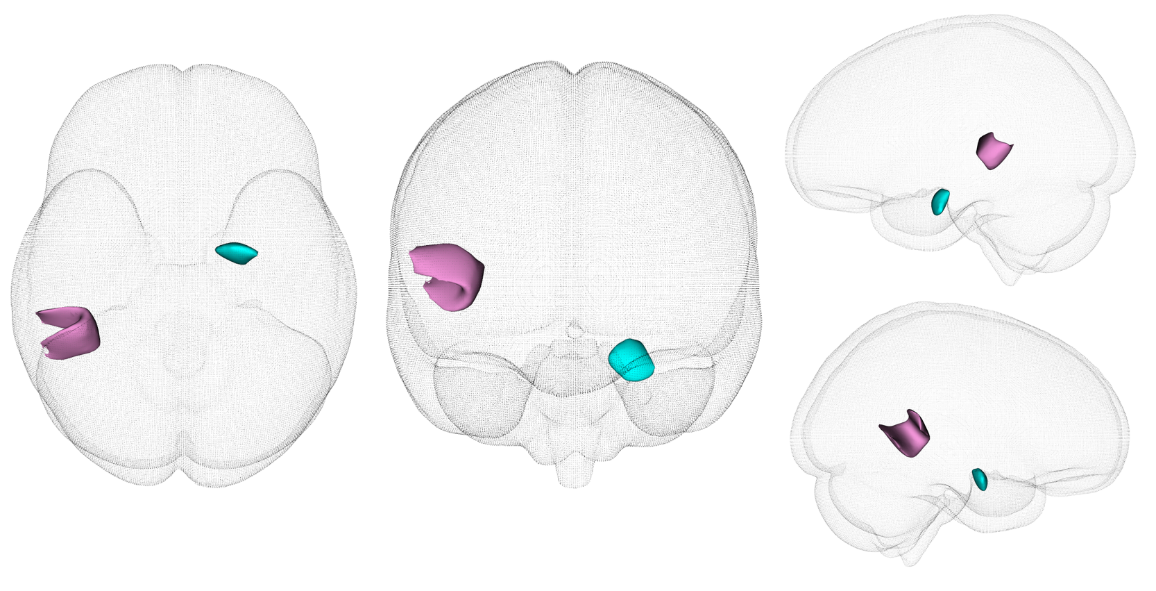


L

R

**Supplementary Experiment 1**

**An independent DTI study on the fibers passing through the right wmSTSbanks**

A total of 15 healthy elderly subjects (mean age 70.6 [SD 6.2] years, 10 females) were enrolled from the local community. DWI (diffusion-weighted imaging) images were obtained with a Siemens 3T Verio scanner (Siemens, Erlangen, Germany) using an EPI (echo planar imaging) sequence with TR (repetition time) = 7600 ms, TE (echo time) = 97 ms, voxel size = 2.3 mm × 2.3 mm × 2.3 mm, FoV (field of view) = 230 mm, matrix size = 122 × 122 and 55 contiguous slices in the axial orientation. The protocol lasted 9 mins including 64 gradient directions with b=1000 s/mm^2^, and a non-diffusion image with b=0 s/mm^2^.

Pre-processing was performed on DWI images including eddy current-induced distortion correction and motion correction using the Functional Magnetic Resonance Imaging of the Brain (FMRIB) Software Library (FSL). Automated reconstruction and labeling of WM, cortical and subcortical regions were performed using Freesurfer on the masked T1-weighted images.^1^ For each participant, a linear registration-based warp was computed from the b0 image to the T1-weighted image (Skull stripping). Then, each individual’s Freesurfer’s segmentation result was transformed based on the invert of the warp from T1 space into the b0 space via FSL.

Constrained spherical deconvolution was used to extract the fiber orientation distribution function (fODF) in each voxel and the anatomically constrained probabilistic tractography (iFOD2) was performed using MRtrix3 to generate five million streamlines with a minimum length of 25 mm,^2,3^ maximum length of 250 mm, step size of 0.5 mm, and 10,000 randomly placed seeds per voxel. The output tracks were cropped at the grey matter and white matter (GM-WM) interface. By generating such a large number of fibers, we were able to investigate a complete set of tracts-of-interest that projected to the hippocampus and amygdala in the majority of the subjects while overcoming the cross-fiber problem during fiber tracking.

Given the whole brain tractography, we investigated the tracts-of-interest that passed through the white matter at the banks of the right superior temporal sulcus (wmSTSbanks) to the hippocampus and amygdala in the same hemisphere, two specific regions that are believed to be targeted by AD. Specifically, we performed an editing operation on the whole brain tractography via specifying the inclusion regions of interest in the hippocampus and amygdala, separately. Then, we only retained the fibers that passed through the right wmSTSbanks, which generated figure e-2. In addition, to investigate which cortical areas are the fibers passing through the right wmSTSbanks ending up with, we specifically chose the tracts-of-interest that passed the right wmSTSbanks from the whole-brain tractography results. Then, we performed a radial search from each streamline endpoint to locate the nearest cortical node with a search distance of 2 mm. The results from the 15 subjects were then combined, generating the fiber densities of each voxel in the right wmSTSbanks that reach to each cortical regions of interest (Figure e-3).

**Supplementary Figure 2 |** Fiber streamlinesthat pass through the wmSTSbanks


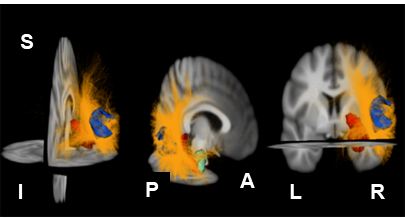


Fiber streamlines in orange, wmSTSbanks in blue, hippocampus in red, and amygdala in cyan

**Supplementary Figure 3** | Cortical regions that can be reached through the right wmSTSbanks


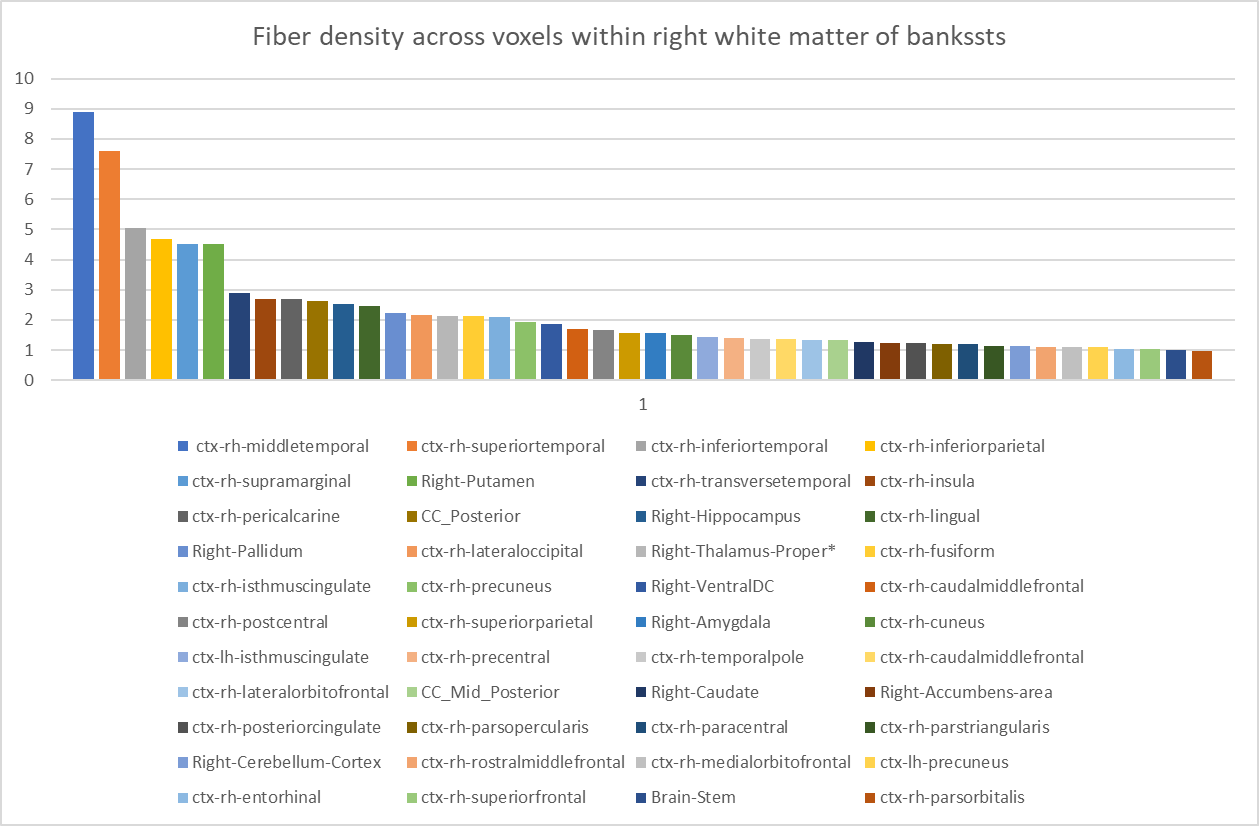


**Supplementary Experiment 2**

**Comparison of the baseline volume of the right wmSTSbanks from the groups with different cognitive stages with the pSCD group (a CLAS study)**

To investigate whether there were alterations in the baseline volume of the right wmSTSbanks in other groups with different cognitive stages compared to the progressive SCD (pSCD) group, from the CLAS database, we selected different cohorts, including 22 stable normal controls (sNC), 16 progressive NCs (pNC) who converted to MCI 7 years later, and 17 stable MCI subjects (sMCI) who were diagnosed as amnestic MCI in both baseline and follow-up, and compared the right wmSTSbanks’s volume of each group with the pSCD group. We analyzed their data in the same way as we did in the main analysis. Therefore, together with the sSCD group included in the main analysis, we separately conducted two-sample *t*-tests for each of the four groups to the pSCD group. First, we found a significant one-way ANOVA result, indicating significant group differences among the five groups (*F* = 3.755, *p* = 0.0064), Second, *post hoc* pairwise comparisons with the pSCD group with Bonferroni corrections (*p* threshold was set to 0.05/4 = 0.0125, which is equal to *p*_corrected_ < 0.05, as four comparisons were conducted) showed a significant difference between sNC and pSCD (*p* = 0.0076) in addition to a significant difference between sSCD and pSCD (*p* = 0.0005). We also found a trend-to-significant difference between sMCI and pSCD (*p* = 0.024 < 0.1/4 = 0.025, which is equal as a *p*_corrected_ < 0.1). Third, the pNC group was found to be in between of the sSCD/sNC/sMCI and pSCD, making it another evidence of early changes in the volume of the wmSTSbanks in the subjects with AD pathology. However, as the pNC subjects can be considered at an even earlier stage with AD pathology than the pSCD, they did not show as much elevated volume of the right wmSTSbanks as the pSCD did, but still considerably higher than the sNC (*p*_uncorrected_ = 0.18, two-sample *t*-test) and sSCD (*p*_uncorrected_ = 0.075, two-sample *t*-test). Finally, as SCD can still be considered as NC, we further combined sNC with sSCD as a stable group without cognitive impairment and tested their mean baseline volume of the right wmSTSbanks against the mean of pNC+pSCD, a progressive group without cognitive impairment, and that of the sMCI group. Similar to the main results, the one-way ANOVA comparing the three groups shows significant difference among these groups (*F* = 6.760, *p* = 0.0016). As shown in Supplementary Figure 4, *post hoc* pairwise comparisons with two-sample *t*-tests showed a significant difference between stable NC/SCD and progressive NC/SCD (*p*_uncorrected_ = 0.0003, *p*_corrected_ < 0.01, Bonferroni correction), as well as a trend-to-significant difference between progressive NC/SCD and sMCI (*p*_uncorrected_ = 0.017, *p*_corrected_ < 0.1, Bonferroni correction).

**Supplementary Figure 4 |** Comparison of the baseline volume at right wmSTSbanks among different groups in CLAS study

*
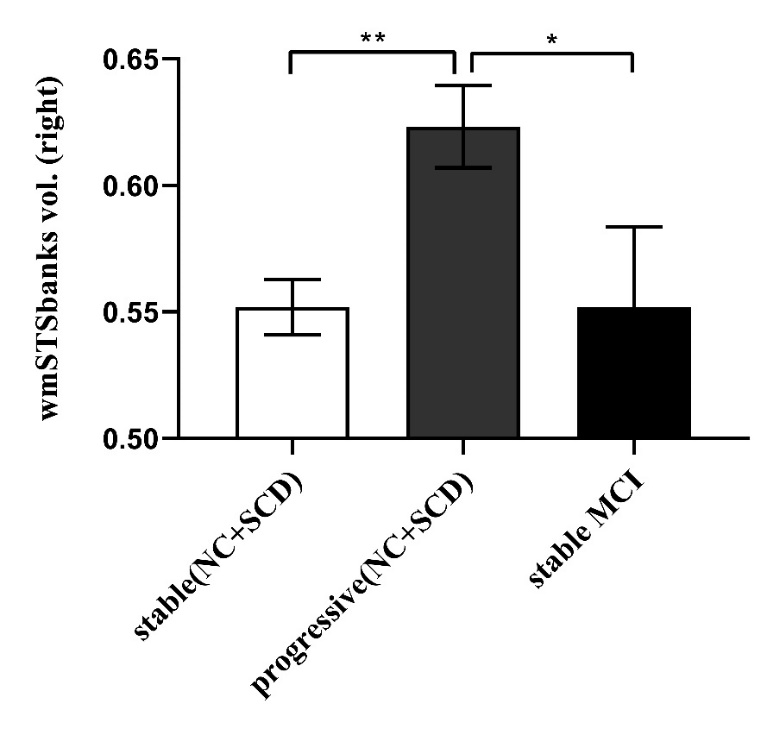
*

A stable group with normal cognitive functions (sNC+sSCD), a progressive group with normal cognitive functions (pNC+pSCD), and a stable group with impaired cognitive functions (sMCI). * shows a trend-to-significant difference (*p*_corrected_ < 0.1, Bonferroni correction with an initial threshold *p* of 0.1/3); ** shows a significant difference (*p*_corrected_ < 0.01, Bonferroni correction with an initial threshold *p* of 0.01/3). Error bars indicate standard errors. The region volume was standardized (no unit).

**Supplementary Experiment 3**

**Comparison of the baseline volume at the right wmSTSbanks between stable normal controls (sNCs) and progressive normal controls (pNCs) (an ADNI study)**

For the purpose of early prediction, we only chose the baseline cognitively normal elderly data (N = 127) and divided them into two groups: sNC (N = 109) and pNC (N = 18), which is similar to the main analysis of SCD subjects (still cognitively normal) and the Supplementary Experiment 2. Although from the ADNI database we could find out a similar cohort namely significant memory concern (SMC) to the SCDs in the main analysis, which was diagnosed by a self-report significant memory concern from the participant but with quantitatively normal cognitive functions (as defined by both the Cognitive Change Index and the Clinical Dementia Rating (CDR) of zeros),^4^ the follow-up time to distinguish progressive SMCs (pSMCs) from stable SMCs (sSMCs) is too short [30.38 months (SD 9.27), ~2.5 years] and their sample size is too small (sSMC, N = 5; pSMC, N = 11). Therefore, we did not include the SMC subjects in this analysis. For the MRI data analysis with FreeSurfer, we took the same procedure with the same parameters. After the volumes of the right wmSTSbanks were extracted, they were standardized by the total white matter volume for each subject. While the pNCs had an elevated mean of baseline wmSTSbanks’ volume [0.62 (SD 0.13), no unit since the region volume was standardized] compared to that of the sNCs 0.59 (SD 0.09), no significant statistical difference was found between them (*p* = 0.24, two-tailed two-sample *t*-test, see Figure 4C in the main text).

**References**

1. Fischl B. FreeSurfer. *Neuroimage* 2012; **62**(2): 774.

2. J-Donald T, Fernando C, Alan C. Robust determination of the fibre orientation distribution in diffusion MRI: non-negativity constrained super-resolved spherical deconvolution. *Neuroimage* 2007; **35**(4): 1459-72.

3. Tournier JD, Mori S, Leemans A. Diffusion tensor imaging and beyond. *Magnetic Resonance in Medicine* 2011; **65**(6): 1532-56.

4. Weiner MW, Veitch DP, Aisen PS, et al. 2014 Update of the Alzheimer's Disease Neuroimaging Initiative: A review of papers published since its inception. *Alzheimer's & dementia : the journal of the Alzheimer's Association* 2015; **11**(6): e1-120.
